# Facilitating Thought Progression to Reduce Depressive Symptoms: Randomized Controlled Trial

**DOI:** 10.2196/56201

**Published:** 2024-11-07

**Authors:** Shai-Lee Yatziv, Paola Pedrelli, Shira Baror, Sydney Ann DeCaro, Noam Shachar, Bar Sofer, Sunday Hull, Joshua Curtiss, Moshe Bar

**Affiliations:** 1 The Leslie and Susan Gonda Brain Science Center Ramat-Gan Israel; 2 Depression Clinical and Research Program Department of Psychiatry Massachusetts General Hospital Boston, MA United States; 3 Department of Psychiatry Harvard Medical School Boston, MA United States; 4 Old Dominion University Norfolk, VA United States

**Keywords:** depression, cognitive neuroscience, facilitating thought progression, FTP, mobile phone, digital health, gamification, depression symptoms, randomized controlled trial, RCT, app, depressive disorder, web-based platforms, effectiveness

## Abstract

**Background:**

The constant rise in the prevalence of major depressive disorder calls for new, effective, and accessible interventions that can rapidly and effectively reach a wide range of audiences. Recent developments in the digital health domain suggest that dedicated online platforms may potentially address this gap. Focusing on targeting ruminative thought, a major symptomatic hallmark of depression, in this study we hypothesized that delivering a digital health–based intervention designed to systematically facilitate thought progression would substantially alleviate depression.

**Objective:**

The study aims to investigate the efficacy of a novel digital intervention on the reduction of depressive symptoms. This intervention was designed as an easy-to-use gamified app specifically aimed to facilitate thought progression through intense practicing of associative, semantically broad, fast, and creative thought patterns.

**Methods:**

A randomized clinical trial was conducted, comparing changes in depression symptoms between participants who used the app in the intervention group (n=74) and waitlist control group (n=27) over the course of 8 weeks. All participants filled out a battery of clinical questionnaires to assess the severity of depression at baseline and 4 and 8 weeks after starting the study. These primarily included the Montgomery-Åsberg Depression Rating Scale (MADRS) and the Patient Health Questionnaire-9 as well as the Positive Affect Negative Affect Scale-Negative Affect Score, Ruminative Response Scale, and Symptoms of Depression Questionnaire. Additional questionnaires were implemented to assess anxiety, positive affect, anhedonia, and quality of life.

**Results:**

The results indicate that across multiple clinical measurements, participants in the intervention group who played the gamified app showed greater and faster improvement in depressive symptoms compared with their waitlist control counterparts. The difference between the groups in MADRS improvement was –7.01 points (95% CI –10.72 to –3.29; *P*<.001; Cohen *d*=0.67). Furthermore, the difference in improvement between groups persisted up to 4 weeks posttrial (MADRS differences at week 12: *F*_49,2_=6.62; *P*=.003; ηp^2^=0.21). At the end of the trial, participants who played the app showed high interest in continuing using the app.

**Conclusions:**

The results demonstrate that a gamified app designed to facilitate thought progression is associated with improvement in depressive symptoms. Given its innovative and accessibility features, this gamified method aiming to facilitate thought progression may successfully complement traditional treatments for depression in the future, providing a safe and impactful way to enhance the lives of individuals experiencing depression and anxiety.

**Trial Registration:**

ClinicalTrials.gov NCT05685758; https://clinicaltrials.gov/study/NCT05685758

## Introduction

### Background

Depressive and anxiety disorders have been on a constant rise as major global disease burdens since 1990 and were among the 10 most common noncommunicable diseases in 2019, even among children as young as 10 years [1]. Major depressive disorder (MDD), which is highly comorbid with anxiety, is characterized by persistent feelings of sadness, loss of interest or pleasure (ie, anhedonia), changes in appetite or weight, disturbances in sleep patterns, fatigue, feelings of worthlessness or guilt, impaired cognitive and social function, and recurrent thoughts of death or suicide [2], altogether making it a highly complex disease.

The global COVID-19 pandemic contributed significantly to the sharp increase in the already-high MDD and anxiety conditions; in 2020, the global number of mental health disorder cases rose significantly, with an additional 76.2 million cases of MDD and 53.2 million cases of anxiety disorder [3]. The COVID-19 pandemic–associated lockdowns and social distancing have led patients to seek and successfully engage with remote psychotherapeutic options, such as online sessions with their therapists, or digital health tools, such as mobile phone apps, for the diagnosis and treatment of depression and anxiety [4]. A recent meta-analysis has demonstrated great efficacy for digital mental health interventions [5], despite their limited face-to-face component and the constant technical support they require. In that manner, digital health has emerged as a promising avenue for addressing mental health challenges in 3 critical aspects: accessibility, retention, and innovation.

### Accessibility

Digital health has the clear benefit of allowing existing treatments a wider reach. For example, telehealth and internet-based approaches that translate existing treatments to online platforms have already been shown to be feasible and as effective as in-person care [5-9]. These remote evidence-based interventions use mobile phone apps as an additional platform for delivering care, encompassing clear advantages given their extensive accessibility, immediacy, low cost, and the ability for patients to use these tools at their convenience without having to wait for mental health professionals to be available [10,11]. In particular, cognitive digital health interventions have attracted considerable attention because of their potential for targeting cognitive processes associated with depression more directly [5,9,12-14]. Digital mental health therapies can significantly improve well-being, potentially overcoming stigma-related barriers to seeking treatment and reaching a much wider population, all while enhancing existing treatments [15].

### Retention

Digital health platforms can amplify retention in favor of mental health causes by harnessing and customizing online gaming features. A review of studies on digital health apps has found that gamification, the inclusion of game elements within nongame contexts, increased motivation and engagement and resulted in improved outcomes [16]. Such interventions leverage the power of technology to deliver not only accessible and cost-effective support but also its power to sustain long-term motivation and engagement for diverse mental health problems [17-19]. Recent studies have found that online gaming platforms incorporating cognitive or behavioral techniques can help alleviate depression [20-22], demonstrating how gaming can complement existing approaches and potentially improve treatment outcomes for various mental health issues [23-25].

### Innovation

The success rate of traditional treatments for MDD, including psychotherapy and pharmacotherapy, remains unsatisfactory: approximately one-third of individuals with MDD do not respond to standard treatments, and high rates of relapse and residual symptoms further highlight the limitations of currently available therapies [26-32]. Furthermore, a large portion (40%-50%) of the population experiencing depression does not even seek treatment [33]. Consequently, there is a clear need for new and innovative solutions, which digital health solutions are well positioned to provide. A central problem with existing treatment options is their focus on symptoms rather than the underlying causes of depression [34,35]. Therefore, although creating gamified online versions of existing treatments may improve accessibility, cost, and engagement, these developments have yet to solve the limited effectiveness of current therapeutic approaches. In other words, beyond successfully implementing remote versions of old treatments, there is an urgent need for remote and gamified solutions that implement innovative interventions and improve treatment outcomes, making life easier for those coping with depression.

### Facilitating Thought Progression

This study evaluated a new app-based intervention developed on the basis of a cognitive neuroscience framework for mood and depression, which we termed *facilitating thought progression* (FTP) [36]. FTP is based on research showing that specific thought patterns are directly and reciprocally connected with mood [37-41]. For example, narrow, slow, and repetitive thinking patterns have been associated with a more negative mood, whereas broad, rapid, and expanding thinking patterns are associated with a more positive mood. The FTP approach postulates that persistent rumination not only dampens mood but over time also leads to structural changes in critical brain areas, such as the hippocampus and the prefrontal cortex [42-44]. Given the consistent findings linking depression and anxiety with ruminative thought patterns (repetitive, circular thinking, sluggish thinking, and narrow semantic representation), an FTP-based intervention would focus on reducing ruminative thoughts by broadening associative scope, reducing mental inhibition, and increasing cognitive flexibility.

In addition, it has been demonstrated that people with depression show a reduced ability for globalization—they exhibit a localized, detail-oriented perspective and are less able to develop a global one. This means that they are limited in their ability to think about things in global terms or in their holistic semantic representation [45,46]. Given the bidirectional relationship between cognitive thought process and mood [35,39,40,47,48], individuals with depression may benefit from cognitive training to facilitate thought patterns that are more broadly associative, more global, and more rapid.

Consistently, our group developed a farm-like mobile phone app game called MoodVille with the aim of providing this form of cognitive training. The app included 5 minigames, and participants needed to complete levels in each of them to earn collectible rewards for their farm development. The therapeutic games were collectively aimed to facilitate thought progression and specifically to enhance cognitive flexibility, associative breadth, creativity, rapid thinking, and globalization. An overview of the app and examples of the levels for each therapeutic game are provided in Figure S1 in Multimedia Appendix 1.

The study aimed to evaluate the efficacy of this FTP-based therapeutic intervention in improving symptoms among patients with MDD.

## Methods

### Participants

The study was a single-blind, randomized controlled trial (RCT) to test the efficacy of a newly developed FTP-based mobile phone app in alleviating symptoms of depression in individuals with MDD. Clinicians evaluating the participants were blinded to their trial condition. Overall, 117 participants aged between 20 and 50 (mean age 32.43, SD 8.5) years were found eligible for the study. Of the 117 participants, 13 (11.1%) from the intervention group and 3 (2.5%) from the waitlist control group (WLC) discontinued either due to technical and administrative reasons (n=5, 4.2%), personal reasons (n=1, 0.8%), or failure to meet minimum game playing requirements (n=10, 8.5%). Therefore, the intervention group consisted of 74 participants, while the WLC group consisted of 27 participants.

### Study Procedure

The study procedures were approved by the Mass General Brigham Institutional Review Board. The study was preregistered (2022P002100). Participants were recruited between October 17, 2022, and May 31, 2023, from online channels, including Craigslist, Facebook (Meta), Instagram (Meta), Reddit, ClinicalTrials.gov, and the recruitment website for research studies conducted in the Mass General Brigham [49]. Interested individuals were first screened over the phone by the study staff. Eligible participants were scheduled to complete a visit with clinical study personnel, where they completed the consent procedure and were evaluated for inclusion and exclusion criteria. The inclusion criteria were meeting the criteria for current MDD per the Mini-International Neuropsychiatric Interview [50] and having mild to moderate depression per the Montgomery-Åsberg Depression Rating Scale (MADRS; MADRS score ≥15 and <35) [51]. The minimum score of 15 was chosen because a cutoff score of 15 to 16 has been shown in 1 study to be the most sensitive for MDD [52], as it falls between mild and moderate severity, suggesting that it is the ideal score to indicate the presence of MDDs. The upper score of 35 was chosen because it indicates the presence of severe depression, which was not identified by the authors as a treatment population and raised safety concerns about participation in a placebo-controlled trial in which the treatment intervention had not yet been validated. Exclusion criteria included the following: meeting the criteria for schizoaffective disorder, bipolar I and II disorder, current posttraumatic stress disorder, panic disorder, obsessive-compulsive disorder that significantly impairs their functioning or lasts >1 hour per day, or personality disorder as long as their MDD diagnosis was primary; having treatment-resistant depression; planning to change treatment regimen (therapy or psychotropic medications) during the 8-week study period; having changed treatment regime (therapy or psychotropic medication) in the 6 weeks before the study initiation; using a computer, internet, or smartphone software–based app for mental health or depression treatment in the 6 weeks before the study; and presence of neurodegenerative diseases or uncorrected visual or dominant-hand motor deficits. Eligible participants were then randomly assigned (3:1) to either the intervention group or the WLC group by a research coordinator. To control for sex at birth and gender-diverse status, blocks of randomized number sequences were used at randomization.

Following randomization, participants in the intervention group began playing the FTP-based mobile app, while WLC participants were informed that they would gain access to the app after an 8-week waiting period. Consistently, study staff contacted the WLC participants at their 8-week mark and offered them a link to download the FTP-based app. During the 8-week study period, participants from both groups were administered web-based weekly questionnaires on depression [53] (Patient Health Questionnaire-9 [PHQ-9]), anxiety [54] (General Anxiety Disorder-7), rumination [55] (Ruminative Response Scale), anhedonia [56] (Snaith-Hamilton Pleasure Scale [SHAPS]), and mood [57] (Positive and Negative Affect Schedule); a monthly quality of life questionnaire [58] (World Health Organization Quality of Life Brief Version); and the Symptoms of Depression Questionnaire [59]. At the end of the study, the intervention group also received a user feedback questionnaire regarding the app itself. All web-based questionnaires were administered and stored via REDCap (Research Electronic Data Capture; Vanderbilt University), a secure web app for surveys and databases. MDD symptoms were evaluated monthly using the MADRS diagnostic questionnaire. Mini-International Neuropsychiatric Interview and MADRS assessments were conducted by clinicians with Master’s, Doctor of Philosophy, or Doctor of Medicine-level qualifications who were blind to group assignment and were extensively trained to administer the assessments.

### Intervention

Intervention arm participants were given a link to download the FTP-based MoodVille app free of charge and were instructed to play the app for at least 4 days per week and 15 minutes per day for 8 weeks. Participants who did not meet the play requirement for >2 weeks over the course of the 8-week trial (ie, missing >25% of minimum play requirements) were discontinued, as per the study’s protocol and consent form. Study staff regularly monitored each participant’s play rate and alerted participants who were close to not meeting their targets. In total, 47 (54%) of the 87 participants in the intervention group were receiving either psychotherapy (n=5, 11%), medication (n=27, 57%), or a combination of both (n=15, 32%). All participants were instructed not to change their treatment regimens during the 8-week study.

The intervention was a farm-like mobile phone app game called MoodVille, and it included 5 minigames, each designed to train 1 of the following cognitive facets: associative breadth, cognitive flexibility, thinking speed, creativity, and global attention (Figure S1 in Multimedia Appendix 1).

### Control Group

The WLC allowed participants to continue their regular MDD treatment protocols, if such existed, and were instructed not to change them for the duration of the 8-week waiting period. A total of 19 participants in the WLC group were receiving either psychotherapy (n=3, 16%), medication (n=8, 42%), or a combination of both (n=8, 42%). Study coordinators contacted the WLC participants at their 8-week mark and offered them a link to download the FTP-based app.

### Primary and Secondary Outcomes

The primary outcomes of the clinical trial were changes in the clinical questionnaires. Questionnaires were filled out each week, and changes were compared between the intervention and the control groups. The secondary outcomes of the clinical trial were postintervention effects, measured through data collected at week 12 of the trial from participants from both groups who extended their engagement. Additional secondary outcomes were evaluated through participants’ ratings of the app, contributing to the assessment of the intervention’s efficacy.

### Data Analytic Strategy

The primary analysis of interest in this study was the effect of the FTP-based app on MDD severity. Our hypothesis was that clinical scores would be significantly improved for the intervention group compared with WLC. Several analyses were conducted to test this hypothesis. First, a 2-way mixed repeated measures ANOVA was run to assess differences between the 2 groups in their clinical scores, which were defined as the outcome variables. In this study, the baseline and fourth and eighth weeks of the clinical trial were used as the time points of reference for the within-subject independent variable, and group allocation was used as the between-subject independent variable. To address sample size differences between the 2 groups, additional randomization analysis was conducted: the 2-way repeated measures ANOVA was performed in multiple iterations (n=1000), in which random data subsampling of participants from the intervention group was performed so that the sample size in each iteration was equal between the intervention and WLC groups. The proportion of significant results out of the number of iterations is reported in the Results section. Second, 1-tailed *t* test analyses tested whether the outcome variable of change in clinical scores from baseline was significantly enhanced for the intervention group compared with the WLC group after 4 weeks as well as after 8 weeks (group and weeks operationalized as independent variables). Third, the clinical benefit of the intervention was assessed by computing the standardized mean difference (SMD) for each assessment tool using Cohen effect size. Finally, to assess the rate of clinical improvement over time, a mixed linear regression analysis was run, estimating the change in each clinical questionnaire’s score over the study’s 8 weeks for both groups. A linear model was built for each questionnaire, in which weeks (1-8) and group (intervention and WLC) were implemented as a fixed effect and participants were implemented as the random effect.

Secondary analyses focused on postintervention data. As participants could continue using the app for an additional 4 weeks, these analyses assessed the long-lasting effect of app use on clinical assessment. In this study, the outcome variable of postintervention clinical data was compared between groups at week 12, while dividing the intervention group into those maintaining high engagement (HE) postintervention and those who maintained low engagement (LE) postintervention (engagement as the independent variable). In addition, analyses focused on participants’ metaexperimental feedback, contributing to assessing the intervention’s efficacy. Finally, exploratory analyses were aimed at examining the intervention’s effectiveness specifically on female players, as the intersection between their prevalence in depression diagnoses and their prevalence in adopting play-like apps positions them to gain maximal benefit from the intervention. This is particularly important given the growing interest in the intersection of mental health and gaming. A recent literary review has shed light on the potential positive impact of video games specifically on women in terms of enhancing cognitive, social, and physical abilities [60]. The latter results are reported in Multimedia Appendix 1.

### Sample Size and Power

A sample size of 100 (75 intervention and 25 WLC) was calculated using G*Power (version 3.1; Heinrich-Heine-Universität Düsseldorf), under the assumption of power of 0.80, for which a 2-tailed *t* test would be able to detect a large effect size (0.8) and a 1-tailed *t* test would be able to detect a moderate effect size (0.57). Aiming to detect a moderate effect size was chosen based on recent findings in online depression treatment research showing a 0.6 effect size in PHQ-9 improvement in internet-based cognitive behavioral therapy (CBT) interventions [61], and corresponding to a recent meta-analysis revealing a mean effect size of 0.67 across internet-based studies [62]. The effect size of 0.57 was chosen to detect a slightly smaller effect within the same expected moderate range.

### Ethical Considerations

The study was ethically approved by the institutional review board of Mass General Hospital (approved protocol number 2022P002100), where the study was conducted. All participants provided informed consent before enrollment and were informed that they could opt out at any time. Data files, including participants’ demographic information (eg, age and gender), clinical measures collected by the clinical team as well as self-administered, and app use data, were kept separate from any identifying information, and all analyses were conducted using deidentified data. No deviations were made from the registered protocol. Participants were reimbursed via monthly payment card deposits or e-checks for the following components: baseline visit (first month only): US $25, visits with a study clinician (once every 4 weeks): US $10, and weekly questionnaires: US $5. Participants in the intervention group received an additional US $35 in the week they completed at least 75% of daily available game levels for 4 different games on at least 4 days of that week. Participants not meeting this weekly use and those in the WLC received US $9 per week. Data collection ended on July 26, 2023.

## Results

### Participants

#### Overview

**Figure 1:**
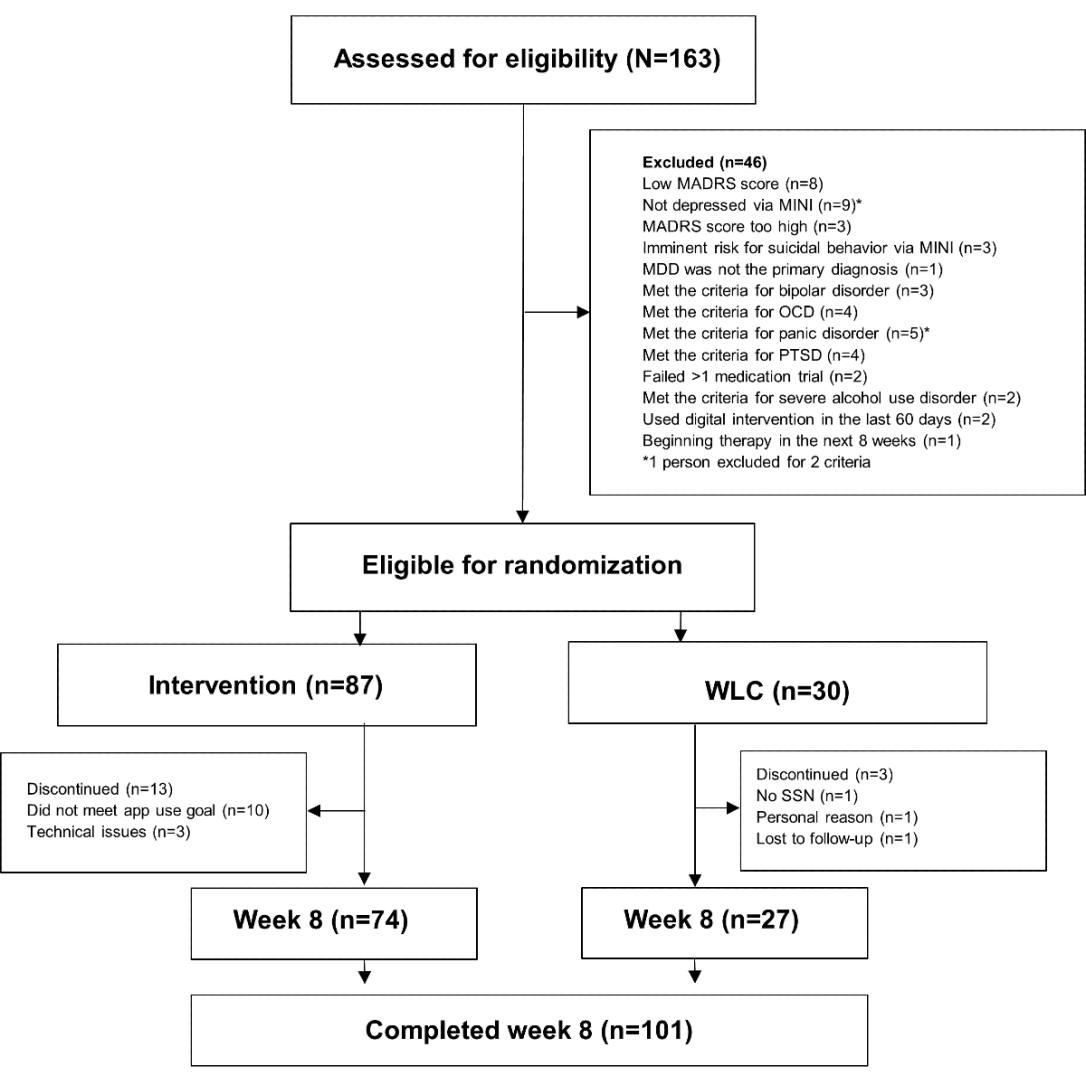
CONSORT (Consolidated Standards of Reporting Trials) table. MADRS: Montgomery-Åsberg Depression Rating Scale; MDD: major depressive disorder; MINI: Mini-International Neuropsychiatric Interview; OCD: obsessive-compulsive disorder; PTSD: posttraumatic stress disorder; SSN: social security number; WLC: waitlist control.

A total of 348 eligible individuals underwent phone screening, and 163 (46.8%) of them met the inclusion criteria for completing a clinical MDD assessment. Of the 163 individuals, 117 (71.7%) were found to be eligible for the study and underwent randomization; of the 117 individuals, 87 (74.4%) were assigned to the intervention group and 30 (25.6%) to the WLC group. The mean age of the participants enrolled in the study was 32.43 (SD 8.5; range 20-50) years, 77.8% (91/117) were women, and 61.5% (72/117) were White. The study was conducted at Mass General Hospital. The primary diagnosis for all 117 participants was MDD, and the mean MADRS score at baseline was 26.97 (SD 4.65). There were no statistically significant differences between the 2 groups on any demographic or clinical variables. During the course of the study, participants were discontinued if they failed to reach the minimum app use requirements for >2 weeks, missing 25% of minimal engagement requirements, as well as if they failed to complete their monthly clinical assessments. Discontinued participants were removed from all analyses. By the end of the study, 13 (14.9%) of the 87 participants from the intervention group and 3 (10%) of the 30 participants from the WLC ended up either discontinuing due to technical and administrative reasons (n=5, 4.2%), personal reasons (n=1, 0.8%), or failure to meet minimum app playing requirements (n=10, 8.5%). Therefore, the intervention group consisted of 73.3% (74/101) participants, while the WLC group consisted 26.7% (27/101) participants (Figure 1). The demographic and clinical characteristics of the 86.3% (101/117) of the participants from both groups who successfully completed the study were not statistically different at baseline from the original groups and are described in Table 1 and Table S1 in Multimedia Appendix 1.

In line with the minimum app playing requirements, which required participants to engage in the app for at least 15 minutes a day for 4 days a week, the mean weekly minutes played was 60.32 (SE 2), showing on-par compliance and little variability across participants.

**Table 1:** Demographic and clinical characteristics at baseline.

			Intervention (n=74)	WLC^a^ (n=27)	*P* value^b^	
**Demographic characteristics**						
	Age (y), mean (SD)		33.22 (8.6)	32.44 (8.53)	.69	
	Female, n (%)		57 (77)	21 (78)	.83	
	**Ethnicity, n (%)**					.56
		American Indian or Alaska Native	2 (2)	0 (0)		
		Asian	12 (16)	8 (30)		
		Black or African American	8 (11)	2 (7)		
		Native Hawaiian or other Pacific Islander	0 (0)	0 (0)		
		White	48 (65)	16 (59)		
		Prefer not to answer	4 (5)	1 (4)		
	University level education, n (%)		58 (78)	18 (67)	.23	
	Married or domestic partners, n (%)		26 (35)	8 (30)	.6	
	**Employment status, n (%)**					.25
		Employed	52 (70)	16 (60)		
		Unemployed	13 (18)	4 (15)		
		Student	9 (12)	7 (26)		
	**Annual household income (US $), n (%)**					.18
		≤19,999	5 (7)	3 (11)		
		20,000-39,999	13 (18)	9 (33)		
		40,000-59,999	13 (18)	3 (11)		
		60,000-79,999	7 (9)	5 (18)		
		80,000-99,999	11 (15)	1 (4)		
		≥100,000	19 (26)	6 (22)		
		Prefer not to answer	6 (8)	0 (0)		
	English as the primary language, n (%)		71 (96)	23 (85)	.06	
**Clinical characteristics**						
	MDD^c^ as the primary diagnosis, n (%)		74 (100)	27 (100)	—^d^	
	**Secondary diagnosis, n (%)**					.40
		Suicide behavior disorder	3 (4)	1 (4)		
		Panic disorder	5 (7)	2 (7)		
		Social anxiety disorder	12 (16)	6 (22)		
		Obsessive-compulsive disorder	0 (0)	2 (7)		
		Alcohol use disorder	2 (3)	0 (0)		
		GAD^e^	21 (28)	11 (41)		
	MADRS^f^ at baseline, mean (SD)		27.53 (4.71)	26.78 (4.13)	.47	
	PHQ-9^g^ at baseline, mean (SD)		15.05 (4.59)	15.70 (3.48)	.51	
	GAD-7 at baseline, mean (SD)		9.27 (5.71)	10.52 (5.73)	.33	
	RRS^h^ at baseline, mean (SD)		55.12 (11.63)	58.96 (7.99)	.07	
	SDQ^i^ at baseline, mean (SD)		141.3 (18.26)	146.7 (14.68)	.17	
	PANAS-NA^j^ at baseline, mean (SD)		23.72 (7.63)	25.93 (7.69)	.19	
	PANAS-PA^k^ at baseline, mean (SD)		17.78 (6.23)	20.15 (8.11)	.12	
	WHO-QoL-BREF^l^ at baseline, mean (SD)		77.86 (10.05)	76.48 (9.63)	.54	
	SHAPS^m^ at baseline, mean (SD)		22.93 (6.31)	22.48 (6.30)	.75	
	Receiving psychotherapy, MDD medication, or both n (%)		42 (56.8)	16 (59.3)	.82	

^a^WLC: waitlist control group.

^b^*P* values indicate the significance level and are derived from either the student *t* test or chi-square test by comparing treatment conditions on binary variables. Data from the originally randomized 117 participants did not differ statistically and are presented in Table S1 in Multimedia Appendix 1.

^c^MDD: major depressive disorder.

^d^Not available.

^e^GAD: General Anxiety Disorder.

^f^MADRS: Montgomery-Åsberg Depression Rating Scale.

^g^PHQ-9: Patient Health Questionnaire-9.

^h^RRS: Ruminative Response Scale.

^i^SDQ: Symptoms of Depression Questionnaire.

^j^PANAS-NA: Positive Affect Negative Affect Scale-Negative Affect Score.

^k^PANAS-PA: Positive Affect Negative Affect Scale-Positive Affect Score.

^l^WHO-QoL-BREF: World Health Organization Quality of Life Brief Version.

^m^SHAPS: Snaith-Hamilton Pleasure Scale.

#### Playing the FTP-Based Game Leads to a Significant Reduction in Negative Clinical Scores Compared With Control Group

**Figure 2:**
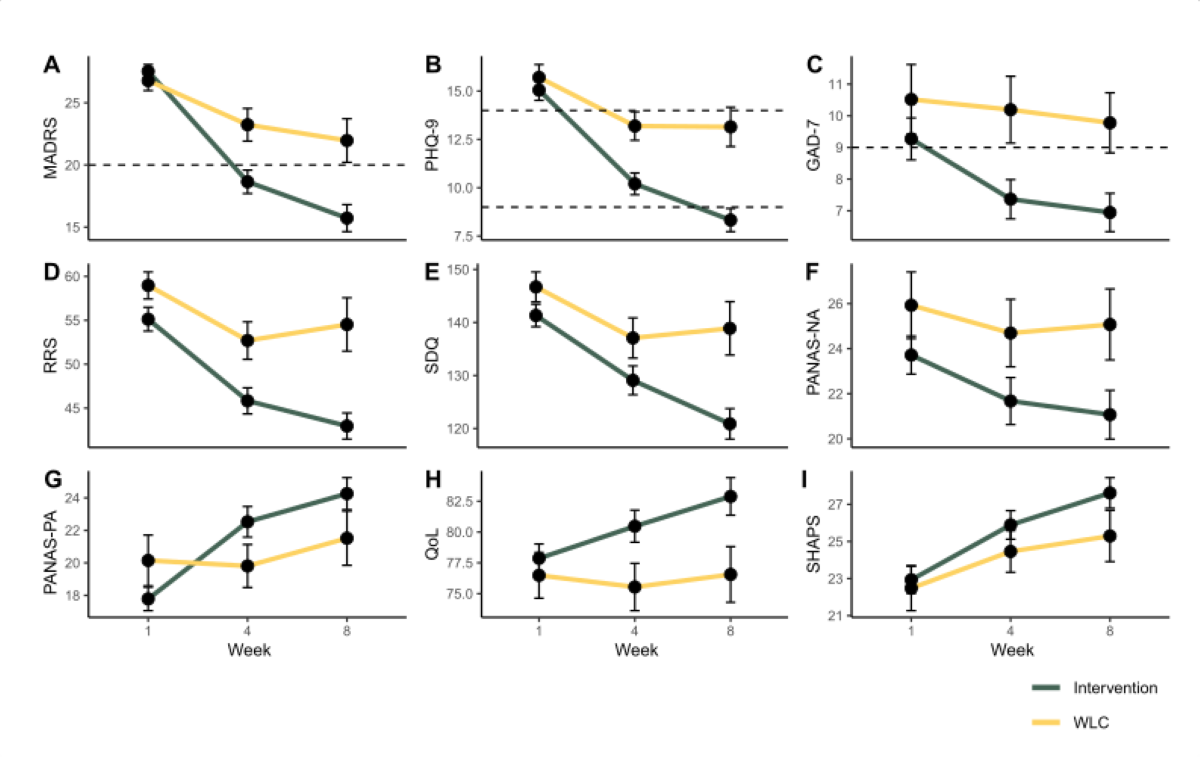
Greater improvement in clinical symptoms following facilitating thought progression–based intervention compared with waitlist control group (WLC). Clinical scores at the first, fourth, and eighth weeks in the intervention and WLC groups. The interaction results of a mixed-effects 3-way repeated measures ANOVA are reported. The proportion of significance in the randomized permutation procedure is indicated in parenthesis. (A) Montgomery-Åsberg Depression Rating Scale (MADRS): F97,2=7.79; *P*<.001 (90% permutation significance). (B) Patient Health Questionnaire-9 (PHQ-9): F95,2=7.17; *P*<.001 (88% permutation significance). (C) General Anxiety Disorder-7 (GAD-7): F95,2=1.75; *P*=.17 (10% permutation significance). (D) Rumination Response Scale (RRS): F97,2=5.67; *P*=.004 (68% permutation significance). (E) Symptoms of Depression Questionnaire (SDQ): F97,2=4.26; *P*=.01 (57% permutation significance). (F) Positive Affect Negative Affect Scale-Negative Affect Score (PANAS-NA): F95,2=1.51; *P*=.22 (6% permutation significance). (G) Positive Affect Negative Affect Scale-Positive Affect Score (PANAS-PA): F95,2=5.75; *P*=.003 (73% permutation significance). (H) World Health Organization Quality of Life Brief Version (WHO-QoL-BREF): F95,2=4.26; *P*=.01 (29% permutation significance). (I) Snaith-Hamilton Pleasure Scale (SHAPS): F97,2=1.08; *P*=.34 (1% permutation significance).

To assess the effectiveness of the intervention compared with the WLC, we conducted a mixed repeated measures ANOVA, separately for each questionnaire, implementing groups as the between-subject variable and time points (ie, baseline, week 4, and week 8) as the within-subject variable. MADRS analysis shows a significant interaction between groups and time points (*F*_97,2_=7.79; *P*<.001), revealing that while there is no difference in MADRS scores between the groups at baseline (intervention: mean 27.52, SD 0.54 and WLC: mean 26.77, SD 0.79; t_99_=0.77; *P*=.44), the intervention group shows significantly lower MADRS scores in the fourth and eighth weeks of the experiment compared with the WLC group (week 4 intervention: mean 18.64, SD 0.94 and WLC: mean 22.37, SD 1.54; t_99_=–2.05; *P*=.04 and week 8 intervention: mean 15.7, SD 1.07, and WLC: mean 21.96, SD 1.75; t_99_=–3.04; *P*=.004). To account for group size differences, an additional permutation test of significance was carried out. For each questionnaire, the intervention group’s participants’ data were subsampled 1000 times to fit the sampling size of the WLC group, and the statistical significance was recalculated. This allowed us to conservatively estimate the proportion of permutations showing a significant interaction effect between groups and time points. In the MADRS analysis, this randomized permutation procedure resulted in a significant interaction in 90% of permutations. These results suggest that the FTP-based intervention effectively reduced clinical symptoms to a greater degree than the WLC. Figure 2 depicts the elaborated results.

**Figure 3:**
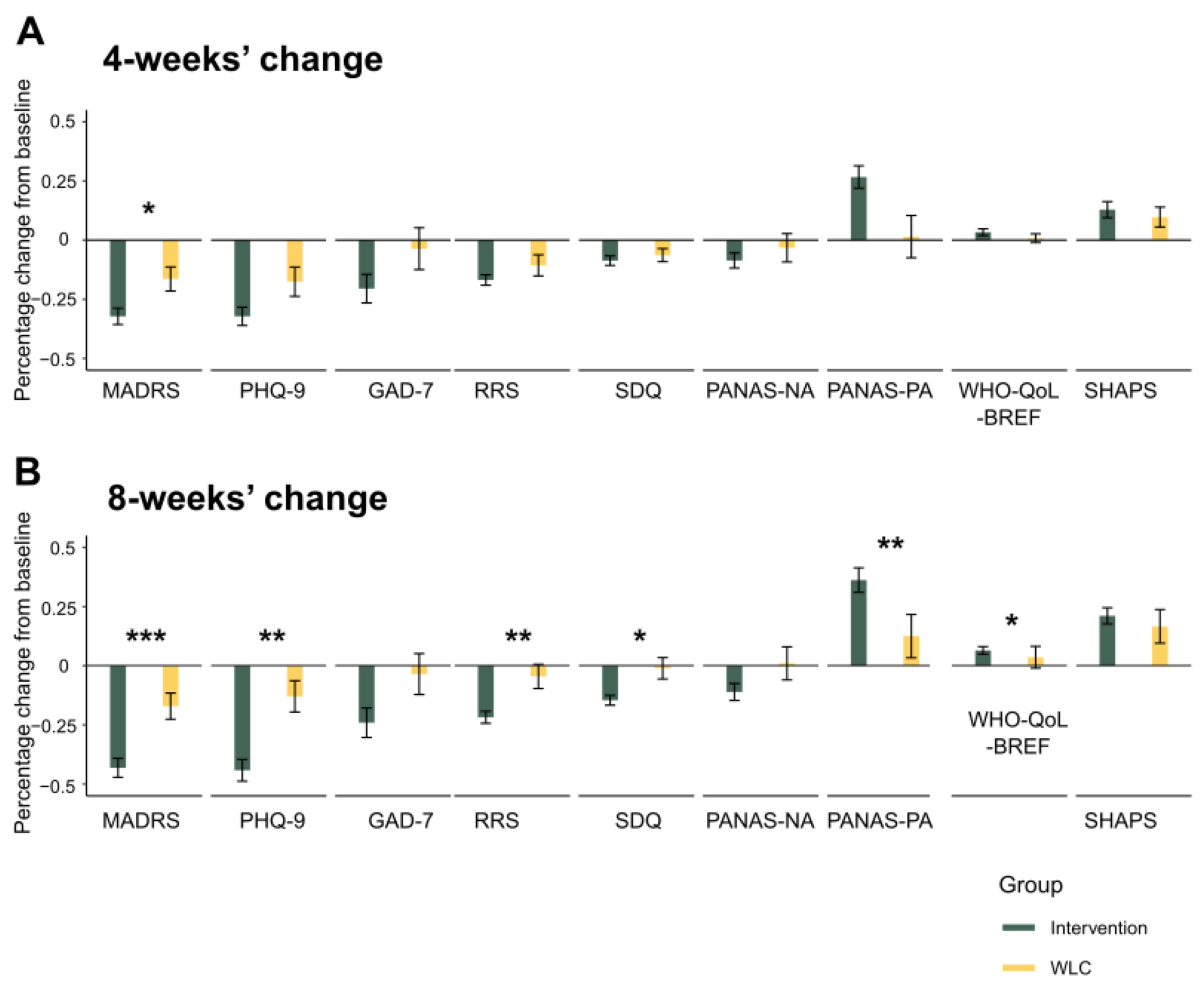
Enhanced improvement in clinical symptoms following the facilitating thought progression (FTP)–based intervention compared with a waitlist control group (WLC). The figure depicts the percentage change from baseline after 4 weeks (A) as well as after 8 weeks (B) of enrolling in the clinical trial in either the FTP-based intervention or the WLC groups. Asterisks denote significant differences in the percentage change from baseline between the intervention and the WLC groups. *P≤.01, **P≤.05, ***P≤.001. Error bars denote SEM. Results are in a normalized format. GAD-7: General Anxiety Disorder-7; MADRS: Montgomery-Åsberg Depression Rating Scale; PANAS-NA: Positive Affect Negative Affect Scale-Negative Affect Score; PANAS-PA: Positive Affect Negative Affect Scale-Positive Affect Score; PHQ-9: Patient Health Questionnaire-9; RRS: Rumination Response Scale; SHAPS: Snaith-Hamilton Pleasure Scale; SDQ: Symptoms of Depression Questionnaire; WHO-QoL-BREF: World Health Organization Quality of Life Brief Version.

Follow-up analyses examined the difference between the groups in their measured clinical change from baseline after 4 weeks as well as after 8 weeks into the trial. The mean change from baseline in the MADRS score after 4 weeks was –8.87 (SE 0.95) in the intervention group and –4.4 (SE 1.4) in the WLC group (difference: –4.47, 95% CI –7.89 to –1.04; *P*=.01) and after 8 weeks was –11.82 (SE 1.12) in the intervention group and –4.81 (SE 1.47) in the WLC group (difference: –7.01, 95% CI –10.72 to –3.29; *P*<.001), indicating a significant difference between the groups in improvement in both time points. Of note, there was no significant difference in the MADRS score at baseline between the groups (difference: 0.75; *P*=.44). Together, these findings suggest the rate of improvement is significantly faster in the intervention group (refer to Figure 3 and Table S2 in Multimedia Appendix 1 for detailed results).

**Figure 4:**
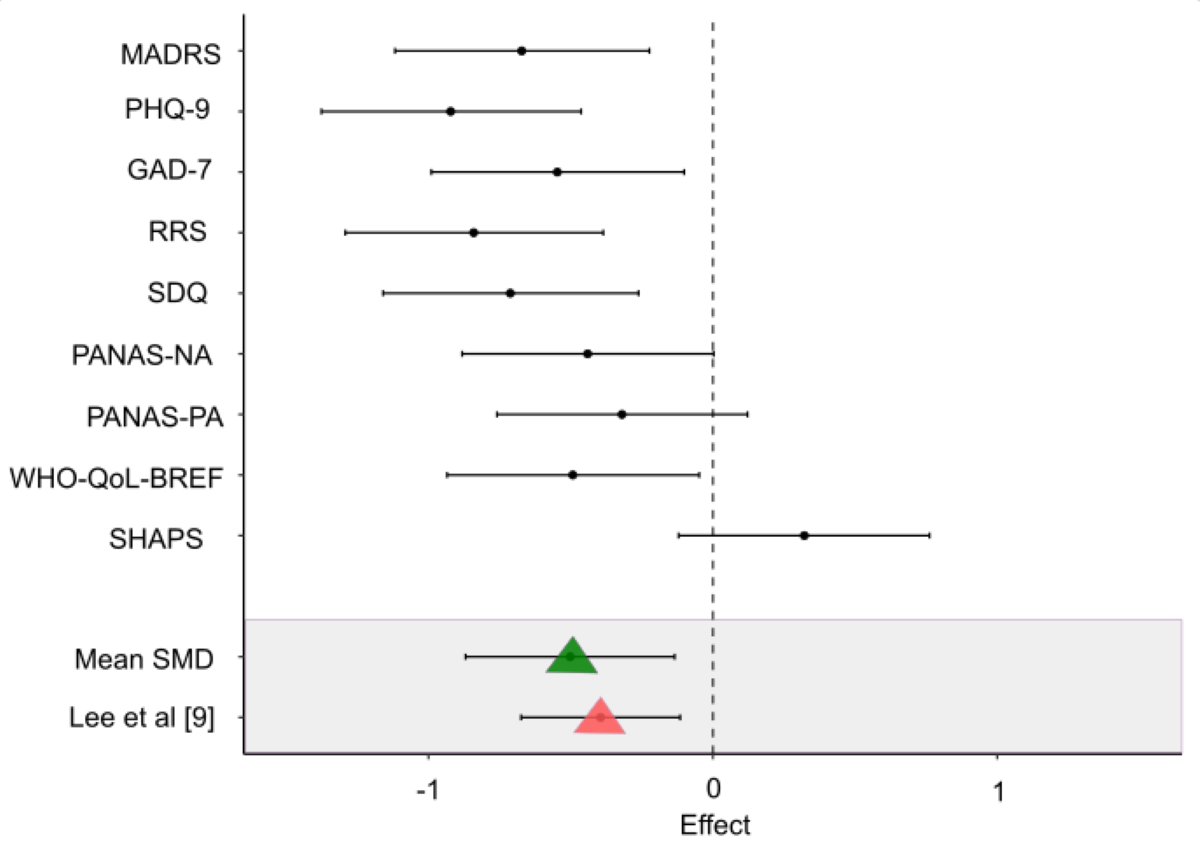
The effect of the facilitating thought progression (FTP)–based app intervention on depression. Dots denote Cohen d effect size. Negative values provide evidence in support of the intervention’s efficiency, while positive values provide evidence in support of waitlist efficiency. Horizontal lines denote CIs. Mean effect sizes for each questionnaire are as follows: Montgomery-Åsberg Depression Rating Scale (MADRS)=−0.67, Patient Health Questionnaire-9 (PHQ-9)=−0.92, General Anxiety Disorder-7 (GAD-7)=−0.54, Rumination Response Scale (RRS)=−0.84, Symptoms of Depression Questionnaire (SDQ)=−0.71, Positive Affect Negative Affect Scale-Negative Affect Score (PANAS-NA)=−0.44, Positive Affect Negative Affect Scale- Positive Affect Score (PANAS-PA)=−0.31, World Health Organization Quality of Life Brief Version (WHO-QoL-BREF)=−0.49, Snaith-Hamilton Pleasure Scale (SHAPS)=0.32. The green triangle denotes the mean effect size across all questionnaires. The pink triangle denotes the mean effect size across all studies reported by Lee et al [9].

Further evaluation of the clinical benefit of the intervention was done by computing the SMD for each assessment tool. This calculation was based on Cohen effect size estimating the difference between the groups at the end of the study (week 8), in line with past studies evaluating the SMD of MDD improvement in mental health digital therapy apps [9]. The results of this analysis support the intervention’s effectiveness over WLC in all assessment questionnaires, with the exception of the SHAPS questionnaire (Figure 4 [9]). The mean effect size across all questionnaires was –0.51, with larger effect sizes of –0.67 in the MADRS and –0.92 in the PHQ-9 questionnaires.

#### Playing an FTP-Based Game Leads to a Significantly Faster Reduction in Negative Clinical Scores Compared With Control Group

To assess the rate of week-by-week clinical improvement, mixed-effects linear models were composed for each clinical questionnaire. These models included all weeks as well as group as fixed effects and participants as a random effect and were found statistically significant for all 9 questionnaires. A significant group effect was found for 6 (66%) out of 9 questionnaires, suggesting that the rates of clinical improvement across these assessments were faster for playing participants compared with WLC (Table 2).

**Table 2:** Rate of improvement in clinical symptoms following facilitating thought progression–based intervention compared with waitlist control group^a^.

Assessment			*β* (SE; 95% CI)		*t* test (*df*)		*P* value		Effect favors intervention significantly		Model *P* values	
**MADRS^b^**												<.001
	Time	–1.24 (0.1; –1.45 to –1.03)		–11.74 (298,5)		<.001		—^c^				
	Group	3.07 (1.33; 0.45 to 5.7)		2.3 (298,5)		.02		Yes				
**PHQ-9^d^**												<.001
	Time	–.64 (0.04; –0.72 to –0.56)		–16.2 (298,5)		<.001		—				
	Group	3.13 (0.85; 1.45 to 4.81)		3.65 (298,5)		<.001		Yes				
**GAD-7^e^**												<.001
	Time	–.29 (0.03; –0.36 to –0.21)		–7.69 (298,5)		<.001		—				
	Group	2.07 (1.03; 0.03 to 4.1)		1.99 (298,5)		.04		Yes				
**RRS^f^**												<.001
	Time	–1.32 (0.9; –1.5 to –1.14)		–14.55 (298,5)		<.001		—				
	Group	8.03 (2.41; 3.29 to –12.76)		3.32 (298,5)		<.001		Yes				
**SDQ^g^**												<.001
	Time	–2.13 (0.28; –2.7 to –1.56)		–7.35 (298,5)		<.001		—				
	Group	10.41 (3.88; 2.76 to 18.05)		2.68 (298,5)		.007		Yes				
**PANAS-NA^h^**												<.001
	Time	–.35 (0.05; –0.51 to –0.27)		–6.27 (298,5)		<.001		—				
	Group	3.46 (1.64; 0.24 to 6.68)		2.11 (298,5)		.03		Yes				
**PANAS-PA^i^**												<.001
	Time	.56 (0.06; 0.44 to 0.68)		9.09 (298,5)		<.001		—				
	Group	–.99 (1.4; –3.74 to 1.75)		–0.71 (298,5)		.47		No				
**WHO-QoL-BREF^j^**												<.001
	Time	.46 (0.11; 0.23 to 0.68)		4.03 (298,5)		<.001		—				
	Group	–4.08 (2.21; –8.44 to 0.27)		–1.84 (298,5)		.06		No				
**SHAPS^k^**												<.001
	Time	.46 (0.05; 0.36 to 0.57)		8.61 (298,5)		<.001		—				
	Group	–1.03 (1.27; –3.53 to 1.46)		–0.81 (298,5)		.41		No				

^a^Mixed-effects general linear models were conducted for each clinical measurement, assessing the rate of clinical symptom change as a function of time (weeks into the clinical trial) and group (playing participants vs waitlist control group). Participants were included in the models as a random variable. Each model’s parameters are reported for the different time and group effects. All significant group effects favor the intervention group. Each model’s *P* values are false discovery rate–corrected for multiple comparisons.

^b^MADRS: Montgomery-Åsberg Depression Rating Scale.

^c^Not available.

^d^PHQ-9: Patient Health Questionnaire-9.

^e^GAD-7: General Anxiety Disorder-7.

^f^RRS: Ruminative Response Scale.

^g^SDQ: Symptoms of Depression Questionnaire.

^h^PANAS-NA: Positive Affect Negative Affect Scale-Negative Affect Score.

^i^PANAS-PA: Positive Affect Negative Affect Scale-Positive Affect Score.

^j^WHO-QoL-BR EF: World Health Organization Quality of Life Brief Version.

^k^SHAPS: Snaith-Hamilton Pleasure Scale.

### Secondary Outcomes

#### Posttrial Follow-Up in Week 12

**Figure 5:**
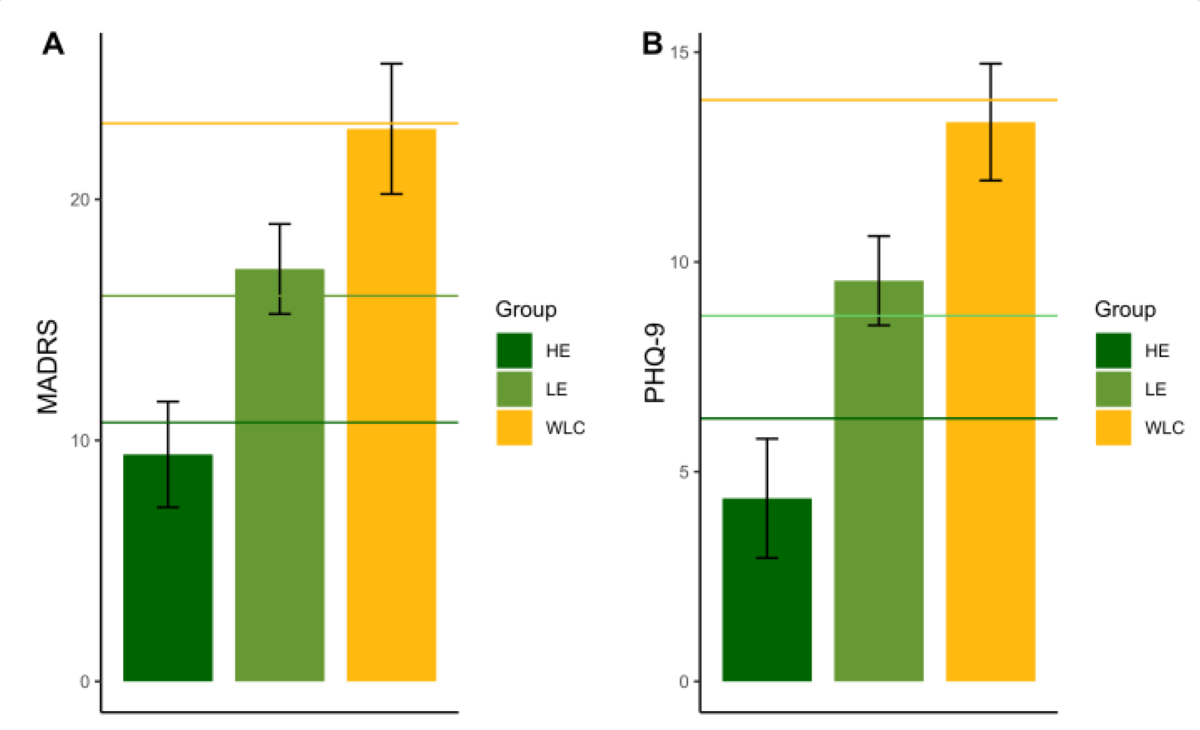
Group differences 1 month after the intervention. (A) Montgomery-Åsberg Depression Rating Scale (MADRS) scores of participants at week 12. (B) Patient Health Questionnaire-9 (PHQ-9) scores of participants at week 12. Bars depict the week-12 scores of high-engagement participants (HE; dark-green bar), low-engagement participants (LE; light-green bar), and waitlist control group (WLC) participants (yellow bar). Horizontal lines depict individual groups’ mean scores at week 8 for reference.

While the study required 8 weeks of engagement, participants could choose to continue for an additional 4 weeks. During this additional phase, participants were compensated for completing the assessment but not for engaging with the app. In total, 90% (58/64) of the participants who were offered the option chose to continue and completed 12 weeks of app use. Though the intervention group contained 73.3% (74/101) of participants who reached the end of week 8, only participants who were recruited before May 2023 were offered to continue playing for another 4 weeks, due to time limitations. In total, 58% (43/74) of the participants from the intervention group and 56% (15/27) of the participants from the WLC group completed a MADRS assessment and filled out self-assessment questionnaires at week 12. In total, 16% (12/74) of the participants from the intervention group maintained the high playing regiment of at least 4 days a week (henceforth HE group), and 42% (31/74) of the participants kept playing but did not maintain regiment-level engagement (ie, missed at least 1 week of playing). These players used the app for 8 (SD 7) minutes throughout the postintervention period (henceforth LE group). In total, 15 (56%) participants from the WLC group underwent MADRS assessment at week 12, out of which 6 (22%) chose to also play the game. These participants played for 24 (SD 10) minutes throughout the 4 (range 12-37) weeks, notably below the minimum 60 minutes per week engagement criteria. The remaining 22% (6/27) of the participants did not play the game at all. Analysis shows that although similar in their baseline MADRS score, participants in the HE group differed from the LE and WLC groups in their clinical scores at week 8, such that the HE group exhibited the lowest MADRS scores at week 8, compared to their LE and WLC group counterparts (*F*_49,2_=7.14; *P*=.002; colored lines in Figure 5A). This difference between the groups was preserved at week 12 (*F*_49,2_=6.62; *P*=.003; colored bars in Figure 5A), as each group maintained an equivalent level to the scores obtained at week 8, suggesting a possible long-lasting effect for the LE group. Similar results were found for the PHQ-9 (week 8: *F*_52,2_=8.48; *P*<.001 and week 12: *F*_52,2_=8.57; *P*<.001; Figure 5B). These results point to the intervention’s possible long-lasting effect as well as suggest that participants for whom the intervention was most effective voluntarily continued to play the app.

#### Subjective Engagement and Benefits

With respect to participant feedback, 47% (35/74) of the participants reported that they saw themselves using the app at least once a week if the app was to be made publicly available. In addition, a high percentage of the participants in the intervention group reported either “agree” or “strongly agree” to the following statements: “Use of this app is likely to decrease low mood and sadness” (34/74, 46%), “The app was easy to use” (70/74, 94%), “Playing this app was fun” (55/74, 74%), as well as “This app is likely to increase awareness of the importance of addressing low mood and sadness” (32/74, 43%) and “This app is likely to increase intentions or motivation to address low mood and sadness” (38/74, 51%), suggesting high subjective perceived benefits. In total, 53% (40/74) of the participants gave the app either 4 or 5 ratings on a 1 to 5 scale, 1 being very negative and 5 being very positive views of the app. The mean score was 3.59, and the median score was 4 (IQR 3-4).

## Discussion

### Principal Findings

This RCT aimed to test the efficacy of a digital health intervention, which was designed to facilitate thought progression, on alleviating symptoms of depression over the course of 8 weeks. This intervention stems from the theoretical grounds showing that flexible, fast, broad, creative, and global thinking patterns are bidirectionally tied with mood. On the one hand, they are dampened among people experiencing negative mood disorders, but on the other hand, practicing and instigating these patterns may improve negative mood [38].

The primary outcomes in this trial show that the rate of clinical improvement in the intervention group was faster, and its overall magnitude was significantly larger compared with that of the WLC. This was true both for several main gold standard measurements for depression, most importantly MADRS and PHQ-9, as well as for General Anxiety Disorder-7, which assessed the improvement in anxiety symptoms. The effect size of the results was on par with previous results found in other existing apps for depression. Secondary outcomes showed that the positive clinical effect persisted for at least 1 month after the trial and that the intervention was perceived as fun, engaging, and beneficial. Overall, these findings demonstrate the efficacy of an FTP-based app in the long-term reduction of depressive symptoms.

Of note, other existing treatments have focused on the existence of maladaptive thought in depression. For example, CBT focuses on identifying negative, irrational, or distorted thoughts and encourages patients to assess their validity and replace them with more beneficial alternatives [63,64]. However, while the CBT intervention aims at teaching strategies to change the content of thought [65,66], the FTP-based approach focuses on changing the structure and pattern of thoughts by having patients engage in cognitive exercises that are independent from thought content. Therefore, the FTP approach is agnostic to the content of the thought or to the level to which one’s thought content corresponds with realistic truth and rather focuses on the structure of thought patterns and how they progress. Therefore, it is likely that an intervention based on the FTP framework could be a complement to CBT.

Beyond its relation to CBT, this study aligns well with the accumulating literature showing sustained beneficial effects of online digital interventions in mental health [11-14]. These found that mobile apps may have the greatest impact on people with mild to moderate depression, which is the audience targeted in this study. These studies also show that video games facilitate cognitive control and multitasking, accompanied by attention-related neural changes, which suggest that digital interventions may lead not only to externally measured improvement but to changes in the underlying mechanisms as well. While the effect of the FTP-based intervention on neural processes remains to be tested, the significant findings in this study corroborate this literature by showing significant improvement in depressive and anxiety symptoms following consistent cognitive training that aims to sustain healthy thinking patterns.

### Theoretical Implications and Future Directions

The main results indicated that participants in the intervention group showed faster and greater clinical improvement compared with WLC. This improvement was further sustained for 4 weeks after the critical period, and overall high compliance was assessed, supporting the efficacy of the intervention and its potential to engage other clinical populations in need. This is the first study showing that an app based on the idea of the FTP framework is associated with improvement of depression. Therefore, altering thought structure may be as important as changing the content of thought. The findings support the notion that practicing faster, broader, and more associative thought patterns may alleviate depression. Future studies may examine whether applying this intervention in parallel to other psychotherapeutic interventions, such as CBT, as suggested earlier, or even classical psychotherapy, boosts their anticipated effect. It is possible that engaging in CBT-like treatments, which focus on rerouting the content of negative thought, conjointly with engaging with the FTP-based digital intervention, which focuses on the structural (eg, breadth) and dynamic-related (eg, pace) aspects of thought, described and tested in this study, may show promising results in targeting depression.

The intervention discussed in this study provides proof of concept for the feasibility of an FTP-based digital intervention to clinically aid populations with depression. Future studies are required to understand how each specific component within the FTP approach impacts depression. For example, fostering broad associative thoughts may have a specific positive effect on rumination reduction, while fast thinking may instate a needed sense of progress. The combination between these elements may also lead to a unique effect on depressive symptoms. Given that this trial evaluated engagement of all 5 therapeutic “minigames,” studies that will further dissociate the FTP approach to its individual therapeutic elements may provide insight into whether different elements can be tailored to different patients with depression for maximal clinical improvement.

### Limitations

This study has several limitations that would require future work to mitigate. First, the experimental design does not allow us to discern whether increased app engagement accelerates the reduction in depressive symptoms. The study required all participants in the intervention group to engage in the FTP-based app for a minimum of 15 minutes daily for 8 weeks, and most participants requested to continue playing for 4 weeks more. However, little variability was observed in the overall duration of engagement, leaving the question open as to whether more extensive engagement would provide additional benefits. Future studies in which participants are free to play as much or as little as they like would help fine-tune the parameters of optimal engagement (eg, daily duration and number of levels solved) that will maximize efficacy in alleviating depression symptoms.

Second, another possible limitation is the cohort’s low ethnic and socioeconomic diversity. Future research should involve better diversifying strategies to improve the ability to conclude efficacy for the broader MDD population.

Third, the study lacks a sham app control group to control for possible placebo effects. For example, while the reported significant changes in MADRS scores in both the intervention and the WLC groups may have resulted from the placebo effects related to being assessed by professional clinicians, the boosted improvement in MADRS in the intervention group may have resulted from an additional placebo effect caused by mere app engagement. A sham app would have controlled for this potential placebo effect, making the study a true double-blind design. Considering this limitation, follow-up examinations evaluated the possibility that our findings were driven by placebo effects, revealing several key insights into why a mere placebo account is unlikely. First, improvement was not observed on all assessment tools, as SHAPS scores did not improve for either group. Second, in an exploratory analysis, it was found that for women, the intervention was more effective as age increased (Multimedia Appendix 1 [67-71]). Together, these findings provide additional support for the specific efficacy of the app for most clinical measurements, though not all, and suggest that its effectiveness may vary more significantly among certain populations. Third, a concern for a placebo effect is generally attributed, in major part, to the possible worsening of symptoms among the WLC, which may eventually account for misattributed beneficial intervention results. In this study, however, the WLC showcased improvement in symptoms, yet it was outperformed by the intervention group, which further supports the app’s effectiveness. Finally, while the RCT did not involve a sham app, the SMD score of all 9 clinical measurements, and specifically the SMD of MADRS, fell well on the high end of effective measures compared with other digital therapeutic RCTs. This analysis suggests that the intervention method developed and studied in this trial is comparable in its magnitude of depressive symptoms reduction to other recently developed online treatments, making a pure placebo account highly unlikely.

### Conclusions

Within the global context of the rising need for innovative solutions that effectively alleviate depression and anxiety, a digital intervention that was designed to facilitate thought progression was found to effectively reduce depressive symptoms across multiple measurements in this study. It was also found to be appealing to patients with MDD, who showed interest in further engagement. These findings support the implementation of FTP-based interventions in future therapeutic efforts or along with other forms of therapeutic intervention, serving the effective facilitation of widely accessible mental health solutions. The findings further bolster the promising potential of digital health solutions to bridge the increasing gap in access to effective treatments for depression and anxiety.

## Appendix

Multimedia Appendix 1 Illustration of the gamified web-based app used by participants in the intervention group. This game includes 5 minigames aimed to facilitate participant’s thought progression.

Multimedia Appendix 2 CONSORT-eHEALTH checklist (V 1.6.1).

## Abbreviations

CBT: cognitive behavioral therapy
FTP: facilitating thought progression
HE: high engagement
LE: low engagement
MADRS: Montgomery-Åsberg Depression Rating Scale
MDD: major depressive disorder
PHQ-9: Patient Health Questionnaire-9
RCT: randomized controlled trial
REDCap: Research Electronic Data Capture
SHAPS: Snaith-Hamilton Pleasure Scale
SMD: standardized mean difference
WLC: waitlist control group

## Data Availability

The datasets generated during and analyzed during this study are available from the corresponding author on reasonable request. The up-to-date app version can be accessed via the MoodBloom app, which is available on the App Store or Google Play Store.
