# Supplementary material for: Facilitating Thought Progression to Reduce Depressive Symptoms: Randomized Controlled Trial
Source: J Med Internet Res. 2024 Nov 7;26:e56201. doi: 10.2196/56201 (PMC11582484; doi:10.2196/56201)
Supplement: Multimedia Appendix 1 [file jmir_v26i1e56201_app1.docx]

Supplementary Material
Figure S1


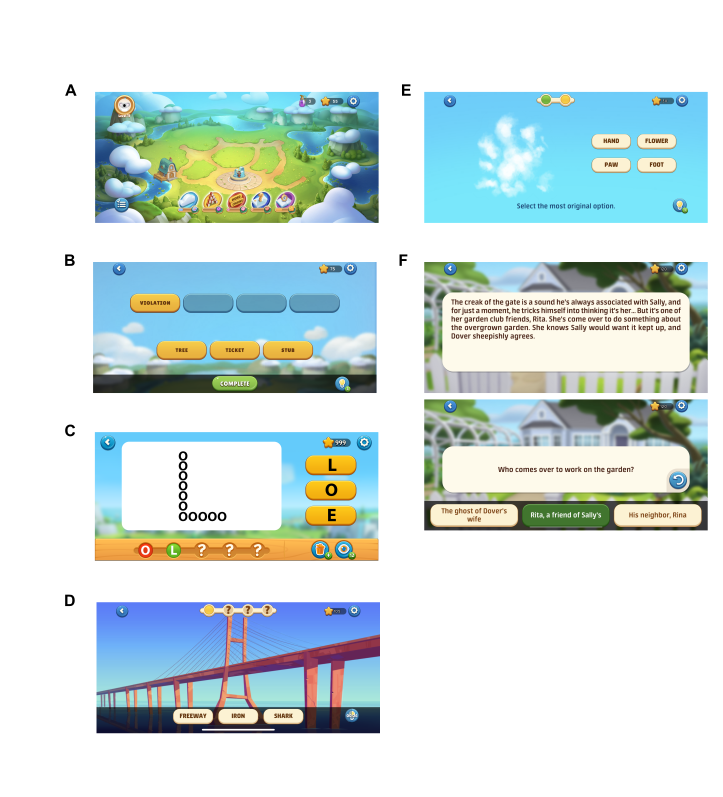


**Figure S1. App illustration and screenshots**. The intervention included five mini-games aimed for facilitating thought progression. **A**. general layout of the game, presenting the five mini-games to play. Participants navigate app-engagement, decide the order of mini-game played, and the number of levels played within each mini-game. **B.** The ‘word-chains’ mini-game. Players are requested to arrange the words in a chained manner, such that each word is associated with the word preceding it and the word following it. This game is aimed to facilitate broad associative thought. **C.** The ‘zoom-out’ game. Players are shown a large letter composed of small letters, and are asked to indicate what the big letter is. This game is aimed to facilitate global attention. **D.** The ‘belong’ mini-game. Players are shown an image of a scene and are asked to choose two words depicting something that could have appeared in the scene, or is semantically related to the scene. This game is aimed to facilitate associative thought. **E.** The ‘clouds’ mini-game. Players are asked to choose the most creative interpretation for the meaning of a given abstract ‘cloud’. This game is aimed to facilitate creative thought. **F.** The ‘speed-read’ mini-game. Players see a short text, which is rapidly presented on screen, and answer a question related to the text. This game is aimed to facilitate fast thinking. Games were implemented within a broader ‘farm’ like game, and collectible points earned at each therapeutic mini-game were used for building and designing the farm.

Table S1

|  | **Intervention (n=87)** | **WLC (n=30)** | **P-value** |
| --- | --- | --- | --- |
| **Demographic Characteristics** |  |  |  |
| Mean Age (±SD) | 32.62 (±8.61) | 31.87 (±8.33) | 0.68 |
| Female (%) | 68 (78.2) | 23 (76.7) | 0.81 |
| Ethnicity (%) |  |  | 0.37 |
| American Indian or Alaska Native | 3 (3.4) | 0 (0) |  |
| Asian | 14 (16.1) | 9 (30) |  |
| Black or African American | 12 (13.8) | 2 (6.7) |  |
| Native Hawaiian or Other Pacific Islander | 0 (0) | 0 (0) |  |
| White | 54 (62.1) | 18 (60) |  |
| Prefer Not to Answer | 4 (4.6) | 1 (3.3) |  |
| University Level Education (%) | 67 (77) | 21 (70) | 0.44 |
| Married/Domestic Partners (%) | 28 (32.2) | 9 (30) | 0.82 |
| Employment Status (%) |  |  | 0.57 |
| Employed | 60 (69) | 19 (63.3) |  |
| Unemployed | 14 (16.1) | 4 (13.3) |  |
| Student | 13 (14.9) | 7 (23.3) |  |
| Annual Household Income (%) |  |  | 0.21 |
| 0 - 19,999 | 7 (8) | 3 (10) |  |
| 20,000 - 39,999 | 15 (17.2) | 10 (33.3) |  |
| 40,000 - 59,999 | 16 (18.4) | 5 (16.7) |  |
| 60,000 - 79,999 | 9 (10.3) | 5 (16.7) |  |
| 80,000 - 99,999 | 11 (12.6) | 1 (3.3) |  |
| 100,000 or above | 21 (24.1) | 6 (20) |  |
| Prefer Not to Answer | 8 (9.2) | 0 (0) |  |
| English as Primary Language (%) | 82 (94.3) | 26 (86.7) | 0.18 |
| **Clinical Characteristics** |  |  |  |
| MDD as Primary Diagnosis (%) | 87 (100) | 30 (100) |  |
| Secondary Diagnosis (%) |  |  | 0.38 |
| Suicide Behavior Disorder | 3 (3.4) | 2 (6.7) |  |
| Panic Disorder | 5 (5.7) | 3 (10) |  |
| Social Anxiety Disorder | 15 (17.2) | 6 (20) |  |
| Obsessive Compulsive Disorder | 0 (0) | 2 (6.7) |  |
| Alcohol Use Disorder | 3 (3.4) | 0 (0) |  |
| Binge-Eating Disorder | 1 (1.1) | 0 (0) |  |
| Generalized Anxiety Disorder | 25 (±28.7) | 13 (±43.3) |  |
| Mean MADRS at Baseline (±SD) | 27.29 (±4.64) | 26.03 (±4.63) | 0.20 |
| Mean PHQ-9 at Baseline (±SD) | 14.79 (±4.52) | 15.41 (±3.82) | 0.51 |
| Mean GAD-7 at Baseline (±SD) | 9.44 (±5.54) | 10.62 (±5.56) | 0.32 |
| Mean RRS at Baseline (±SD) | 55.54 (±11.71) | 58.03 (±8.46) | 0.22 |
| Mean SDQ at Baseline (±SD) | 141.15 (±18.4) | 145.48 (±14.87) | 0.25 |
| Mean PANAS-NA at Baseline (±SD) | 23.93 (±7.06) | 25.76 (±7.44) | 0.24 |
| Mean PANAS-PA at Baseline (±SD) | 18.43 (±6.45) | 20.21 (±7.89) | 0.23 |
| Mean WHO-QoL-BREF at Baseline (±SD) | 77.95 (±9.93) | 77.14 (±9.83) | 0.70 |
| Mean SHAPS at Baseline (±SD) | 23.60 (±6.39) | 22.03 (±7.38) | 0.27 |
| Receiving Psychotherapy and/or  MDD Medication (%) | 47 (54) | 18 (60) | 0.57 |

**Table S1. Demographic and clinical characteristics at baseline**. p = significance value, derived from either student’s t-test or chi-square (*χ*2 ) by comparing treatment conditions on binary variables.
Note: the final analysis was conducted on 73 and 27 completers from the intervention and WLC groups, accordingly.

Table S2

| Assessment | Mean change- intervention | | Mean change- WLC | |
| --- | --- | --- | --- | --- |
| MADRS | 0-4 | -8.87±0.95^***♰^ | 0-4 | -4.4±1.4^**^ |
|  | 0-8 | -11.82±1.12^***♰^ | 0-8 | -4.81±1.47^**^ |
| PHQ-9 | 0-4 | -4.85±0.58^***^ | 0-4 | -2.76±0.93^**^ |
|  | 0-8 | -6.68±0.71^***♰^ | 0-8 | -2.55±1.03^*^ |
| GAD-7 | 0-4 | -1.90±0.56^**^ | 0-4 | -0.38±0.82^n.s^ |
|  | 0-8 | -2.21±0.59^***^ | 0-8 | -0.74±0.86^n.s^ |
| RRS | 0-4 | -9.29±1.22^***^ | 0-4 | -6.3±2.46^*^ |
|  | 0-8 | -12.04±1.43^***♰^ | 0-8 | -4.44±2.55^n.s^ |
| SDQ | 0-4 | -12.24±2.85^***^ | 0-4 | -9.34±3.86^*^ |
|  | 0-8 | -20.56±3.06^***♰^ | 0-8 | -7.81±5.02^n.s^ |
| PANAS-NA | 0-4 | -2.04±0.76^**^ | 0-4 | -0.84±1.42^n.s^ |
|  | 0-8 | -2.61±0.88^**^ | 0-8 | -0.85±1.65^n.s^ |
| PANAS-PA | 0-4 | 4.74±0.84^***♰^ | 0-4 | 0.3±1.59 |
|  | 0-8 | 6.47±0.95^***♰^ | 0-8 | 1.37±1.53^n.s^ |
| WHO-QoL-BREF | 0-4 | 2.6±1.11^*^ | 0-4 | -0.61±1.43^n.s^ |
|  | 0-8 | 5.01±1.27^***♰^ | 0-8 | 0.07±2.04^n.s^ |
| SHAPS | 0-4 | 2.95±0.78^***^ | 0-4 | 2.19±0.97^*^ |
|  | 0-8 | 4.73±0.8^***^ | 0-8 | 2.81±1.29^*^ |

**Table S2: Improved clinical symptoms following FTP-based intervention compared with WLC**. The table depicts for each assessment its mean change from baseline after 4 weeks, as well as after 8 weeks of enrolling in the clinical trial to either the FTP-based intervention or the WLC groups. Asterisks denote significant changes from baseline. ♰= significant differences in mean change from baseline between the intervention and the WLC groups. GAD-7= General Anxiety Disorder-7; MADRS= Montgomery–Åsberg Depression Rating Scale; PANAS-NA= Positive Affect Negative Affect Scale- Negative Affect Score; PANAS-PA= Positive Affect Negative Affect Scale- Positive Affect Score; PHQ-9= Patient Health Questionnaire-9; RRS= Rumination Response Scale; SHAPS= Snaith-Hamilton Pleasure Scale; SDQ= Symptoms of Depression Questionnaire; WHO-QoL-BREF= World Health Organization Quality of Life Brief Version; WLC= Waitlist Controls

*Female Spotlight*
In addition to our main findings, we were interested in assessing whether FTP-based app use was more effective given specific demographic conditions. Considering that females compose a larger proportion of humans experiencing depression and anxiety [66], [67], [68], and are also more likely to seek help [69], [70], it was important to understand the relation between their demographic attributes and the intervention. Indeed, selectively for female players, we find a significant correlation between age and clinical improvement, such that the older the player was, there was a greater reduction in MADRS and PHQ-9 scores between baseline and week 8 (MADRS: r= -0.33, p=0.01; PHQ-9: r= -0.33, p=0.01). These correlations did not emerge for male participants, nor did they emerge in the WLC.
